# F^18^-FDG PET-CT Findings in Juvenile-Onset Polyarteritis Nodosa: A First Series and Literature Review

**DOI:** 10.3390/jcm14093012

**Published:** 2025-04-27

**Authors:** Clément Triaille, Sebastien Benali, Julie Barsalou, Elie Haddad, Victor Kokta, Raphael Kraus, Raymond Lambert, Marie-Paule Morin, Kathryn Samaan, Sophie Turpin, Jean Jacques De Bruycker

**Affiliations:** 1 Division of Pediatric Immunology and Rheumatology, CHU Sainte-Justine, Department of Pediatrics, University of Montreal, Montreal, QC H3T1C5, Canada; clement.triaille@uclouvain.be (C.T.);; 2 Pôle de Pathologies Rhumatismales Systémiques et Inflammatoires, Institut de Recherche Expérimentale et Clinique, Université Catholique de Louvain, Brussels 1200, Belgium; 3 Radiology-Medical Imaging, CHU Sainte-Justine, University of Montreal, Montreal, QC H3T1C5, Canada; 4 Pathology, CHU Sainte-Justine, University of Montreal, Montreal, QC H3T1C5, Canada; 5 Nuclear Medicine-Medical Imaging, CHU Sainte-Justine, University of Montreal, Montreal, QC H3T1C5, Canada

**Keywords:** juvenile polyarteritis nodosa, PET-CT, vasculitis, CNO, CRMO, MRI

## Abstract

**Objectives**: To investigate the findings associated with juvenile polyarteritis nodosa (PAN) on F18-FluoroDeoxyglucose (FDG), positron emission tomography combined with computed tomography (PET-CT). **Methods**: Patients diagnosed with juvenile PAN (onset <18 years) who underwent a PET-CT at diagnosis (before therapy) were enrolled. PET-CT images were systematically analyzed to identify abnormal findings associated with PAN. In addition, a systematic literature review was performed to identify previously published cases. **Results**: Six patients with biopsy-confirmed PAN were identified (age at onset 10–17 years). PET-CT was abnormal in all patients. Patchy muscular and subcutaneous FDG uptake with a symmetric distribution in the lower limbs was present in 4/6 patients. Increased FDG uptake in large arteries was found in 1/6 patients. FDG-avid bone lesions were identified in 2/6; additional MRI and bone biopsy results were consistent with chronic non-infectious osteomyelitis (CNO). Unspecific inflammatory findings (medullar and lymphoid organs hypermetabolism) were present in 6/6; these were the only abnormalities present in 2/6 patients. We found this pattern of PET-CT muscular involvement to differ from juvenile dermatomyositis and septic emboli (*n* = 7 and 2 patients, respectively). In addition, we identified four previously published cases of juvenile PAN investigated with PET-CT: one with FDG-avid muscular and subcutaneous foci, one with increased uptake in large arteries, and two with nonspecific signs (lymphoid organs hypermetabolism). **Conclusions**: This is the first series of juvenile PAN investigated with PET-CT. Diffuse, patchy hypermetabolic foci in the muscular and subcutaneous tissue of the lower limbs were the most common findings. These features should lead to suspicion of PAN. Further research is needed to assess the diagnostic value of PET-CT in PAN.

## 1. Introduction

Juvenile polyarteritis nodosa (PAN) is a rare systemic vasculitis (estimated annual frequency ~1/10^6^ in Sweden [1]), with onset before 18 years. PAN predominantly affects medium- and small-sized arteries, leading to intramural inflammatory infiltration, fibrinoid necrosis, and endovascular thrombi [2]. Although this phenotype may result from germline or somatic monogenic defects or chronic infectious diseases [3,4,5], detailed pathophysiology remains indeterminate in the majority of sporadic, idiopathic cases.

PAN is considered in many clinical contexts: fever/inflammatory syndrome of unknown origin, vasculitis rash or subcutaneous nodules, severe musculoskeletal pain, or specific organ involvement (i.e., renal, digestive, genital, nervous system). PAN diagnosis in children relies on demonstration of small- or medium-vessel vasculitis on tissue biopsy, or vessel abnormalities on imaging (typically conventional angiography or magnetic resonance imaging (MRI)) [6]. Recently, interest has grown for complementary radiological modalities such as F18-FluoroDeoxyglucose (FDG), positron emission tomography combined with computed tomography (PET-CT) in adult-onset PAN [7]. Thus, the most frequent feature in the single published cohort was a diffuse, patchy hypermetabolic signal in skin and muscles (a pattern called ‘leopard skin’ appearance). In juvenile-onset PAN, description of PET-CT findings is limited to scarce case reports [8,9,10,11].

Here, we collected the first series of juvenile-onset PAN investigated by PET-CT, and performed a literature search of previously published cases to provide an overview of PET-CT findings in juvenile-onset PAN.

## 2. Materials and Methods

### 2.1. Patients

This is a single-center retrospective observational study performed at Centre Hospitalier Universitaire Sainte-Justine (CHUSJ), Montréal. This study was approved by the Research Ethics Board of CHUSJ (REB#3251). Inclusion criteria were the following: (i) diagnosis of PAN at CHUSJ based on EULAR/PReS classification criteria [6], (ii) onset before 18 years, and (iii) investigation with 18F-Fluorodeoxyglucose (FDG), positron emission tomography with computed tomography (PET-CT) at diagnosis, before immune suppressive therapy. Patients not fulfilling EULAR/PReS criteria or receiving immunosuppressive therapy before PET-CT were excluded. Clinical, biological, histological, and radiological data (from PET-CT ± corresponding magnetic resonance imaging (MRI)) were collected.

### 2.2. PET-CT

All patients fasted (including discontinuation of dextrose-containing intravenous solutions) for at least four hours prior to 18Fluorodeoxyglucose administration. The blood glucose level was verified prior to injection to ensure values ≤ 8 mmol/L. PET acquisitions were initially performed after injection of 3.5 MBq/kg (range: 37–444 MBq). Imaging was performed between 65 and 85 min after F-18-FDG administration. Imaging was performed using a Philips Gemini 16 time-of-flight PET-CT scanner. The acquisition duration was 2.5 min/FOV. CT parameters for attenuation correction were as follows: 120 kVp, pitch 0.813, rotation time 0.5 s, slice thickness 5 mm, and collimation 16 × 1.5 mm. For whole-body scanning, the CT tube current (mAs) was adjusted according to weight (≤45 kg: 22 mAs; 46–67 kg: 33 mAs; 68–90 kg: 44 mAs; >91 kg: 66 mAs). PET images were acquired in three-dimensional mode and reconstructed using a row-action maximum likelihood algorithm (3 iterations, 33 subsets). No children needed or received sedation for the purpose of PET imaging. The maximum standardized uptake value (SUVmax) was determined from the weight and injected activity using irregular regions of interest.

Maximum intensity projection (MIP) and combined attenuation corrected (CTAC) and non-attenuation corrected (NAC) PET/low-dose CT images were analyzed by expert nuclear medicine specialists (T.S.; L.R.). The following features were evaluated: (i) presence of muscular or fusiform muscular FDG uptake and its distribution; (ii) presence of cutaneous/subcutaneous FDG uptake; (iii) increased FDG uptake in large vessels; (iv) medullar, spleen and lymph nodes metabolism; (v) bone or articular FDG uptake.

We also collected additional imaging performed contemporaneously with PET-CT to enhance the characterization of findings on PET-CT.

### 2.3. Literature Search

We performed a PubMed and Embase search using the following terms: “pediatric/juvenile/childhood onset polyarteritis nodosa” AND “positron emission tomography/PET”. All articles were individually reviewed for the extraction of clinical and radiological data.

### 2.4. PET-CT in Non-PAN Patients

We searched the in-house CHUSJ database of PET-CT with the keyword “myositis” to identify patients with muscle involvement and a diagnosis other than PAN. PET-CT was performed in the same manner as for PAN patients. All cases were reviewed by an expert nuclear medicine specialist (T.S.).

### 2.5. Statistical Analyses

Only descriptive statistics were used. Data are expressed as the median (range) for continuous variables, or as proportions for categorical variables.

## 3. Results

### 3.1. Cohort Description

Six PAN patients were included in the study. Clinical and laboratory data are available in Table 1. The median age at onset was 15 years (range 10–17 years). PET-CT was performed before any immunosuppressive therapy, after a reported symptom duration of 3 to 16 weeks. All patients presented with general malaise, and 5/6 reported prolonged or recurrent fever. Weight loss was confirmed in 2/6. Cutaneous lesions occurred in 6/6 (painful subcutaneous nodules in 6/6, livedo in 3/6, skin ulceration in 1/6), arthralgia/myalgia (predominant in the lower limbs) in 6/6, arthritis or extremity edema in 3/6, and testicular pain in 2/4 males. No patient suffered from elevated blood pressure or renal or digestive involvement. The median peak CRP at onset was 99.5 mg/L (range 52.2–224 mg/L). Antinuclear antibodies (ANA), antineutrophil cytoplasmic antibodies (ANCA), and creatine kinase (CK) were tested as normal in all individuals. Three patients tested negative for deficiency in adenosine deaminase type 2 (DADA2) (the others were not tested). Skin biopsy confirmed necrotizing vasculitis in all patients.

### 3.2. PET-CT (±MRI) Findings in Juvenile Onset PAN

**Figure 1:**
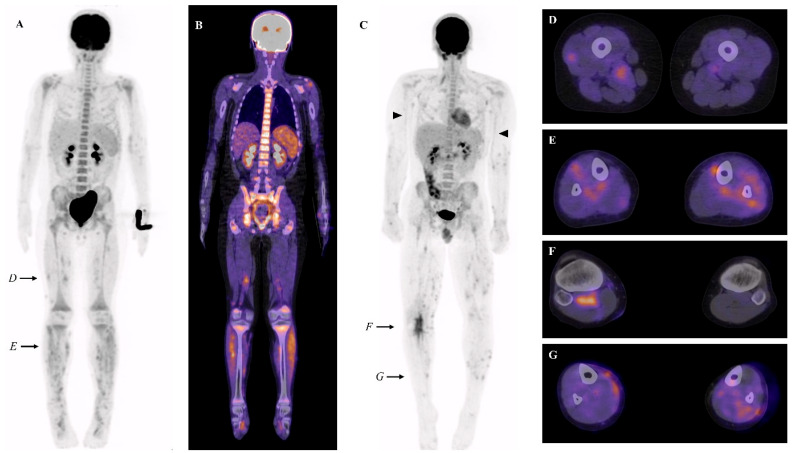
(**A**,**B**) Maximal intensity projection (MIP) (**A**) and representative fused FDG PET-CT coronal view (**B**) of patient 1, showing diffuse hypermetabolic foci in the lower limbs. (**C**) MIP FDG PET-CT of patient 2, showing diffuse hypermetabolic foci in the lower limbs, vascular uptake in both brachial arteries (black arrowhead), and the right popliteal region. (**D**–**G**) Fused FDG PET-CT transverse views showing foci of increased muscular and subcutaneous FDG uptake in patients 1 and 2 (levels of the cross-sectional views are shown by black arrows in (**A**,**C**)).

PET-CT findings of each patient are summarized in Table 1. We found focal heterogeneous muscular FDG uptake in 4/6 patients, with a symmetric distribution (Figure 1A–G). In the same four patients, cutaneous/subcutaneous hypermetabolic foci were also present (Figure 1G). These findings were predominant in the muscles and skin of the lower legs. When numerous, a combination of these abnormalities can result in the so-called ‘leopard skin’ sign (Figure 1A). The intensity of the muscular and cutaneous/subcutaneous hypermetabolism was moderate (SUVmax ranging from 2.2 to 4.7). In addition, increased FDG uptake in large vessels (brachial arteries) was found in 1/6 patients (Figure 1C).

**Figure 2:**
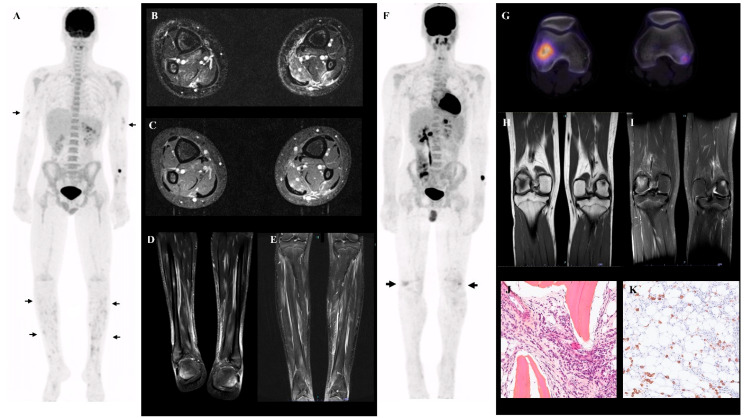
(**A**) FDG PET-CT MIP of patient 3, showing patchy hypermetabolic foci in the limbs, predominantly in the legs (arrows). (**B**–**D**) Axial and coronal MRI of the lower legs of patient 3, showing patchy and diffuse muscular and subcutaneous hypersignal (T2FS sequence in (**B**,**D**), T1 FS in (**C**)). (**E**) Axial MRI of the lower legs of patient 4, showing patchy and diffuse muscular and subcutaneous hypersignals (T1 FS). (**F**) FDG PET-CT MIP of patient 4, demonstrating increased bone uptake in both lateral femoral condyles (thick arrows). (**G**) Fused transverse FDG PET-CT views of the bone lesions in the lateral femoral condyles, with underlying lytic lesions. (**H**–**I**) MRI imaging showing T1 hypointense (T1 TSE sequence in (**H**)), and T2 hyperintense (T2 SPAIR sequence in (**I**)) aspects of the bone lesions. (**J**,**K**) Bone biopsy (right femoral condyle) of patient 4, demonstrating plasmocytic infiltrate and mild medullary fibrosis (Hematoxylin eosin staining in (**J**) and anti-CD138 staining in (**K**) (brown)).

MRI images of the lower limbs, contemporaneous (within 7 days) to PET-CT, were acquired in two patients (patients 3 and 4), both positive for the aforementioned PET-CT findings (Figure 2A). In both, MRI confirmed diffuse, patchy T2-hyperintense signal localized in the muscles and subcutaneous tissue (Figure 2B–E).

Two patients (2 and 4) also displayed bone abnormalities. Patient 4 had three hypermetabolic bone lesions (both femoral lateral condyles and sternum (Figure 2F–G and Supplementary Figure S1A)), with an SUVmax of 5.5. The right lateral femoral condyle lesion showed moderate lysis on CT. On MRI, the lesions were T1 hypointense and T2 hyperintense (Figure 2H–I). Biopsy was suggestive of chronic aseptic osteomyelitis (plasmocytic infiltrate and mild medullary fibrosis, Figure 2J–K). The second patient with abnormal bone PET-CT was found to have a hypermetabolic (SUVmax 5.1) pelvic bone lesion (Supplementary Figure S1B).

We also found nonspecific, inflammation-related findings: hypermetabolic lymphadenopathies in 4/6 and splenic/medullary/thymic hypermetabolism in 6/6 (Figure 1B). These were the only abnormalities present in 2/6 patients. We found no association between clinical or biological features and PET-CT patterns.

### 3.3. Literature Review

We identified four cases of juvenile-onset PAN with descriptions of PET-CT findings published in the literature (Table 2). One patient (8 years old) displayed ‘leopard skin’ sign on PET: diffuse hypermetabolic nodules in the soft tissues of arms and legs [8]. FDG uptake in the arterial walls of the arms and legs was reported in a second patient (9 years old) [9]. An additional two patients had nonspecific findings on PET-CT: hypermetabolic signal in the spleen and bone marrow, or FDG-uptake in lymphadenopathies and intestine [10,11]. Combining published cases with our cohort, we found increased FDG uptake in the subcutaneous or muscular tissue of limbs in 5/10 patients, in the large arteries in 2/10, and in bones in 2/10. Nonspecific, inflammation-related PET-CT findings (hypermetabolic spleen, and/or bone marrow and/or lymphadenopathies) were present in all patients. These were the only PET-CT abnormalities present in 4/10 patients.

### 3.4. PET-CT Findings in Other Conditions

**Figure 3:**
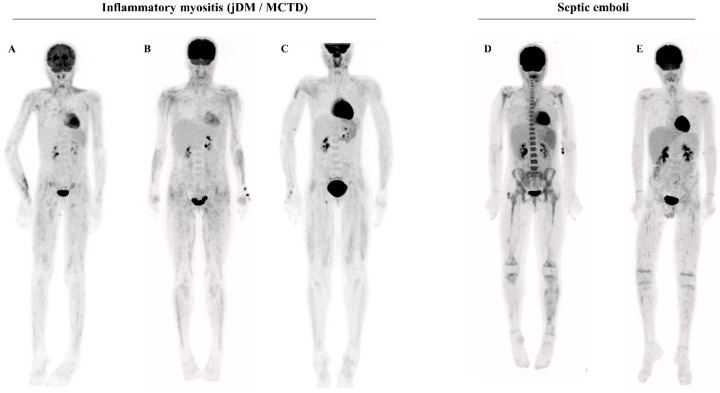
(**A**–**C**) Illustrative examples of PET-CT findings in juvenile dermatomyositis (jDM) or mixed connective tissue disorders (MCTD): symmetrical, diffuse, linear increased muscular uptake affecting the limbs, more marked proximally than distally. No cutaneous/subcutaneous lesions. Anti-MDA5 in (**A**), anti-Ro52 in (**B**), and anti-SRP in (**C**). (**D**,**E**) Illustrative examples of PET-CT findings in patients with septic emboli (Staphylococcus Aureus bacteremia in the context of bacterial endocarditis in (**D**), during a stem cell transplantation for leukemia in (**E**)). Non-symmetrical random regions of uptake in soft tissues of the lower extremities (arrows), including the right greater trochanter bursa in patient (**D**) (arrowhead).

To assess whether the muscular and cutaneous/subcutaneous heterogeneous FDG uptake could be differentiated from muscle inflammation found in other conditions, we searched the local CHUSJ PET-CT database. We compared the PET-CT of PAN patients with that of patients with other inflammatory myositis (*n* = 7) (idiopathic dermatomyositis or myositis of mixed connective tissue disorder), or septic muscular emboli (*n* = 2). The PET-CT of inflammatory myositis displayed the following differences: (i) an absence of cutaneous/subcutaneous FDG uptake; (ii) a more proximal and homogeneous, linear distribution of the muscular FDG uptake (Figure 3A–C). The muscular PET-CT findings in the patients with septic emboli displayed no robust difference from PAN (Figure 3D–E).

## 4. Discussion

We report the first series of juvenile-onset PAN patients evaluated with PET-CT. We describe multiple findings, ranging from diffuse, patchy tracer uptake in muscles ± subcutaneous tissue, to hypermetabolic large-size arterial walls, to nonspecific inflammation-related changes. On MRI (*n* = 2), the same pattern of diffuse heterogeneous involvement of the muscles and subcutaneous fat was found.

Diagnosis of PAN in children is highly challenging. There is no validated diagnostic test or criteria to confirm a diagnosis of PAN. A consensus exists on a set of classification criteria, which requires, as a mandatory finding, the following: necrotizing vasculitis in a small/medium-sized artery on biopsy or angiographic abnormalities (in addition to other signs) [6]. Yet, the yield of angiographic imaging is usually low in children, possibly because most have PAN without organ involvement [12,13]. Conventional angiography and MR angiography in children may also have side effects or require sedation. Therefore, the utility of alternative imaging techniques is relevant to investigate.

The value of PET-CT in diagnosing large-vessel vasculitis (i.e., Takayasu disease, giant cell arteritis) is well established in children and adults [14,15]. By contrast, there is a paucity of data regarding its possible usefulness in PAN, a vasculitis predominantly involving medium- and small-sized arteries. The first cohort of adult patients (*n* = 10, median age 67 years) has been recently published [7]. The authors reported findings comparable to ours: increased FDG uptake in muscles was present in 7/10, and in large-sized arteries in 4/10. Two out of ten patients displayed only non-specific PET-CT findings. Bone hypermetabolism was not reported. Our data demonstrate that muscle involvement is also the most frequent specific finding on PET-CT in juvenile-onset PAN.

Interestingly, we found hypermetabolic bone lesions (in addition to soft-tissue involvement) in 2/6 PAN patients in our cohort. MRI and biopsy of one lesion were suggestive of chronic non-bacterial osteomyelitis (CNO). To the best of our knowledge, CNO has been previously associated with other systemic vasculitis (Takayasu arteritis or ANCA-associated vasculitis), but has only been reported once in the context of PAN [16]. As PAN patients frequently have diffuse pain and are not systematically investigated with dedicated imaging techniques, bone involvement may have been underdiagnosed in this condition.

Importantly, PET-CT findings must be interpreted in their clinical context, as other conditions have been reported with a similar pattern of “leopard sign” in a few adult case reports: dermatomyositis, sarcoidosis myopathy, VEXAS (vacuoles, E1 enzyme, X-linked, autoinflammatory, somatic) syndrome, and hemopathies [17,18,19]. In our series, we found that the PET-CT aspect of juvenile-onset PAN differed from classic dermatomyositis, but we added septic muscular emboli as a possible mimicker.

Several limitations should be considered in the interpretation of our data. First, the retrospective design and sample size prevent direct extrapolation of using PET-CT to confirm a suspected diagnosis of PAN. On the other hand, our data might directly help clinicians to recognize PAN-suggestive findings on a PET-CT performed in the context of a broad differential diagnosis (i.e., in a patient with fever, diffuse pain, and weight loss). For instance, in our cohort, PET-CT was abnormal in two patients two weeks before the typical rash appeared and was biopsied. Another limitation pertains to the specificity of the pathological muscular tracer uptake: we compared our PAN cohort with other conditions reported to have similar PET-CT aspects. Yet, this comparison was performed on small numbers, using qualitative assessment only, and without blinded evaluation. Finally, the sensitivity of PET-CT to detect signs suggestive of PAN also deserves further investigation: in fact, only nonspecific inflammatory signs were found in 4/10 patients (our series + published cases) [10,11]. This is also in line with the recently published adult series [7]. Thus far, it is unclear if the patients with only nonspecific findings differ from the others in terms of clinical activity, tissue involvement, disease duration, and/or stage.

In conclusion, this is the first series to describe PET-CT findings in juvenile-onset PAN. PET-CT was abnormal in all individuals. The most common specific sign was heterogeneous FDG uptake in muscular and cutaneous/subcutaneous tissue (‘leopard skin’ pattern). Our data suggest that PET-CT may be a useful investigation in a patient with suspected PAN. Larger, prospective studies are required to investigate the added value of PET-CT for diagnosing PAN, as well as its potential use as a marker of disease activity and response to therapy.

## Figures and Tables

**Table 1:** PET-CT findings and clinical and biological features in the cohort.

Patient n°		1	2	3	4	5	6
**Clinical and biological features**	Age at onset (years)	10	17	16	15	15	15
	Time between reported symptoms onset and PET-CT	3 weeks	8 weeks	3 weeks	12 weeks	3 weeks	16 weeks
	Prolonged fever	Y	N	Y	Y	Y	N
	Generalized weakness, malaise	Y	Y	Y	Y	Y	Y
	Weight loss	Y	Y	N	N	N	N
	Cutaneous lesions	Y	Y	Y	Y	Y	Y
	Details on cutaneous lesions	Livedo racemosa, subcutaneous nodules	Subcutaneous nodules	Subcutaneous nodules	Subcutaneous nodules	Livedo, subcutaneous nodules	Livedo, subcutaneous nodules
	Myalgia, arthralgia	Y	Y	Y	Y	Y	Y
	Arthritis/extremities swelling	Y	N	Y	N	Y	N
	Testicular involvement	Y (pain, normal ultrasound)	N	N	Y (scrotal ulceration)	NA	NA
	Renal involvement	N	N	N	N	N	N
	Hypertension	N	N	N	N	N	N
	Digestive involvement	N	N	N	N	N	N
	Peak CRP at onset (mg/l)	224	106	80	153	93	52.2
	CK (reference values: 30–200 UI/L)	40	91	19	113	25	29
	Cytopenia	N	N	N	Yes	Anemia	N
	ANCA	negative	negative	negative	negative	negative	negative
	ANA	negative	negative	negative	negative	negative	negative
	ADA2 deficiency	not tested	not tested	not tested	negative (genetic screening)	negative (genetic screening)	negative (enzymatic activity)
	Confirmation of PAN on biopsy	Y (skin)	Y (skin)	Y (skin)	Y (skin)	Y (skin)	Y (skin)
	Treatment	Steroids, IVIG	Steroids	Steroids	Steroids, Mycophenolate mofetil	Steroids and colchicine	Steroids and colchicine
**PET-CT findings**	Abnormal focal muscular uptake	Y	Y	Y	Y	N	N
	Abnormal focal subcutaneous uptake	Y	Y	Y	Y	N	N
	Large vessels uptake	N	Y	N	N	N	N
	Bone marrow and spleen hypermetabolism	Y	Y	Y	Y	Y	Y
	Lymph nodes hypermetabolism	Y (inguinal and popliteal)	Y (axillary, inguinal and popliteal)	Y (popliteal right, inguinal left)	Y (axillary, inguinal and popliteal)	Y (inguinal)	Y (popliteal right)
	Additional hypermetabolic focus	N	Bone (*n* = 1)	N	Bone (*n* = 3)	N	N
	SUV_max_ of muscle/subcutaneous tissue lesions	2.2	4.7	2.6	NA	NA	NA

ADA2: adenosine deaminase 2; ANA: antinuclear antibodies; ANCA: antineutrophil cytoplasm antibodies; CK: creatine kinase; CRP: C-reactive protein; IVIG: intravenous immunoglobulins; PAN: polyarteritis nodosa; PET-CT: positron emission tomography combined with computed tomography. SUV: standardized uptake value.

**Table 2:** PET-CT findings and clinical and biological features of patients previously described in the literature.

Patient		Shimizu et al., 2016 [8]	Pijl et al., 2020 [9]	Ropers et al., 2020 [10]	Jasper et al., 2010 [11]
**PET-CT findings**	Abnormal focal muscular and subcutaneous uptake	Y	N	N	N
	Abnormal focal (subcutaneous) uptake	Y	N	N	N
	Large vessels uptake	N	Y	N	N
	Increased FDG uptake in lymph nodes, spleen, bone marrow, thymus	Y	Y	Y	Y
	Additional hypermetabolic focus	N	N	Intestine (diffuse)	N
	SUV_max_ of muscle/subcutaneous tissue lesions	NA	NA	NA	NA
**Clinical features**	Age at onset (years)	8	9	NA	NA
	Time between reported symptoms onset and PET-CT (weeks)	NA	NA	NA	NA
	Prolonged fever	Y	Y	NA	NA
	Generalized weakness	NA	Y	NA	NA
	Weight loss	NA	NA	NA	NA
	Cutaneous lesions	Y	N	NA	NA
	Details on cutaneous lesions	subcutaneous nodules	N	NA	NA
	Myalgia, arthralgia	Y	Y	NA	NA
	Arthritis/extremities swelling	Y	N	NA	NA
	Testicular	NA	N	NA	NA
	Renal	N	N	NA	NA
	Hypertension	N	N	NA	NA
	Digestive	N	Y	Y	NA
	CRP at onset (mg/l)	14	307	NA	NA
	CK (30–200 UI/L)	NA	NA	NA	NA
	cytopenia	NA	NA	NA	NA
	ANCA	NA	NA	NA	NA
	ANA	NA	NA	NA	NA
	DADA2	NA	NA	NA	NA
	Confirmation of PAN on biopsy	Y (skin)	N	NA	NA

ANA: antinuclear antibodies; ANCA: antineutrophil cytoplasm antibodies; CK: creatine kinase; CRP: C-reactive protein; DADA2: deficiency in adenosine deaminase 2; IVIG: intravenous immunoglobulins; PAN: polyarteritis nodosa; PET-CT: positron emission tomography combined with computed tomography. SUV: standardized uptake value.

## Data Availability

Data are available upon reasonable request addressed to the corresponding author.

## Supplementary Materials

The following supporting information can be downloaded at: https://www.mdpi.com/article/10.3390/jcm14093012/s1, Supplementary Figure S1: **A**–**B**. Fused PET FDG-CT axial views showing FDG-avid bone lesion in the sternum (**A**, patient 4) and pelvic bone (**B**, patient 2) (white arrows).

## References

[1] Mossberg M., Segelmark M., Kahn R., Englund M., Mohammad A.J. (2018). Epidemiology of primary systemic vasculitis in children: A population-based study from southern Sweden. Scand. J. Rheumatol..

[2] Hernández-Rodríguez J., Alba M.A., Prieto-González S., Cid M.C. (2014). Diagnosis and classification of polyarteritis nodosa. J. Autoimmun..

[3] Elkan P.N., Pierce S.B., Segel R., Walsh T., Barash J., Padeh S., Zlotogorski A., Berkun Y., Press J.J., Mukamel M. (2014). Mutant adenosine deaminase 2 in a polyarteritis nodosa vasculopathy. N. Engl. J. Med..

[4] Guillevin L.M., Lhote F.M., Cohen P.M., Sauvaget F.M., Jarrousse B.M., Lortholary O.M., Noël L.-H.M., Trépo C.M. (1995). Polyarteritis nodosa related to hepatitis B virus A prospective study with long-term observation of 41 patients. Medicine.

[5] Beck D.B., Ferrada M.A., Sikora K.A., Ombrello A.K., Collins J.C., Pei W., Balanda N., Ross D.L., Cardona D.O., Wu Z. (2020). Somatic Mutations in *UBA1* and Severe Adult-Onset Autoinflammatory Disease. N. Engl. J. Med..

[6] Ozen S., Ruperto N., Dillon M.J., Bagga A., Barron K., Davin J.C., Kawasaki T., Lindsley C., Petty R.E., Prieur A.M. (2006). EULAR/PReS endorsed consensus criteria for the classifi-cation of childhood vasculitides. Ann. Rheum. Dis..

[7] Fagart A., Machet T., Collet G., Quéméneur T., Ben Ticha R., Verstraete M., Le Gouellec N., Demailly F., Rousselin C. (2022). Fluorodeoxyglucose positron emission tomography–computed tomography findings in a first series of 10 patients with polyarteritis nodosa. Rheumatology.

[8] Shimizu M., Inoue N., Mizuta M., Ikawa Y., Yachie A. (2016). Leopard skin appearance of cutaneous polyarteritis nodosa on^18F^fluorodeoxyglucose positron emission tomography. Rheumatology.

[9] Pijl J.P., Kwee T.C., Legger G., Peters H.J., Armbrust W., Schölvinck E., Glaudemans A.W. (2020). Role of FDG-PET/CT in children with fever of unknown origin. Eur. J. Nucl. Med..

[10] Ropers F.G., van Mossevelde R.M.P., Bleeker-Rovers C.P., van Velden F.H.P., van Assema D.M.E., Adam J.A., Lam M.G., Tolboom N., de Geus-Oei L.F., Frings V. (2020). Evaluation of FDG-PET/CT Use in Children with Suspected Infection or Inflammation. Diagnostics.

[11] Jasper N., Däbritz J., Frosch M., Loeffler M., Weckesser M., Foell D. (2010). Diagnostic value of [18F]-FDG PET/CT in children with fever of unknown origin or unexplained signs of inflammation. Eur. J. Nucl. Med..

[12] Eleftheriou D., Dillon M.J., Tullus K., Marks S.D., Pilkington C.A., Roebuck D.J., Klein N.J., Brogan P.A. (2013). Systemic polyarteritis nodosa in the young: A single-center experience over thirty-two years. Arthritis Rheum..

[13] Ozen S., Anton J., Arisoy N., Bakkaloglu A., Besbas N., Brogan P., García-Consuegra J., Dolezalova P., Dressler F., Duzova A. (2004). Juvenile polyarteritis: Results of a multicenter survey of 110 children. J. Pediatr..

[14] Slart R.H., Nienhuis P.H., Glaudemans A.W., Brouwer E., Gheysens O., van der Geest K.S. (2023). Role of (18)F-FDG PET/CT in Large Vessel Vasculitis and Polymyalgia Rheumatica. J. Nucl. Med..

[15] Soliman M., Laxer R., Manson D., Yeung R., Doria A.S. (2015). Imaging of systemic vasculitis in childhood. Pediatr. Radiol..

[16] Malochet-Guinamand S., Fan A., Perrey A., Soubrier M. (2024). Apremilast successfully treats cutaneous polyarteritis nodosa associated with SAPHO syndrome. Rheumatology.

[17] Oueriagli S.N., El Asraoui L., Sahel O.A., Benameur Y., Doudouh A. (2023). Snow Leopard Appearance of Subcutaneous Panniculitis such as T-cell Lymphoma on 18F-FDG PET/CT. Mol. Imaging Radionucl. Ther..

[18] Fagart A., Quemeneur T., Collet G., Demailly F., Rousselin C. (2023). A “Leopard Man” Aspect on 18 F-FDG PET/CT Revealing a VEXAS Syndrome. Clin Nucl Med..

[19] Fayad F., Duet M., Orcel P., Liote F. (2006). Systemic sarcoidosis: The “leopard-man” sign. Jt. Bone Spine..

