# Supplementary material for: F18-FDG PET-CT Findings in Juvenile-Onset Polyarteritis Nodosa: A First Series and Literature Review
Source: J Clin Med. 2025 Apr 27;14(9):3012. doi: 10.3390/jcm14093012 (PMC12072537; doi:10.3390/jcm14093012)
Supplement: Supplementary file 1 [file jcm-14-03012-s001.zip › jcm-3549944-supplementary.pdf]

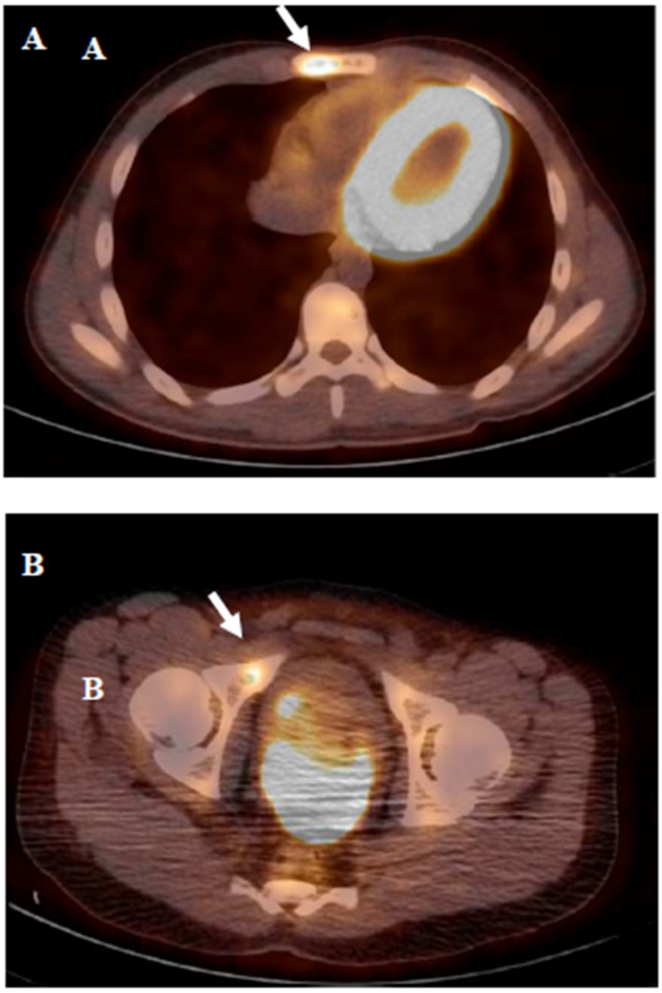

Supplementary Figure S1: A-B. Fused PET FDG-CT axial views showing FDG-avid bone lesion in the sternum (**A**, patient 4) and pelvic bone (**B**, patient 2) (white arrows).
